# Novel Insights into the Echinoderm Nervous System from Histaminergic and FMRFaminergic-Like Cells in the Sea Cucumber *Leptosynapta clarki*

**DOI:** 10.1371/journal.pone.0044220

**Published:** 2012-09-06

**Authors:** Luke A. Hoekstra, Leonid L. Moroz, Andreas Heyland

**Affiliations:** 1 Friday Harbor Laboratories, University of Washington, Seattle, Washington, United States of America; 2 Department of Biology, Indiana University, Bloomington, Indiana, United States of America; 3 The Whitney Laboratory for Marine Bioscience, University of Florida, Gainesville, Florida, United States of America; 4 Department of Neuroscience, University of Florida, Gainesville, Florida, United States of America; 5 Integrative Biology, University of Guelph, Guelph, Ontario, Canada; University of Gothenburg, Sweden

## Abstract

Understanding of the echinoderm nervous system is limited due to its distinct organization in comparison to other animal phyla and by the difficulty in accessing it. The transparent and accessible, apodid sea cucumber *Leptosynapta clarki* provides novel opportunities for detailed characterization of echinoderm neural systems. The present study used immunohistochemistry against FMRFamide and histamine to describe the neural organization in juvenile and adult sea cucumbers. Histaminergic- and FMRFaminergic-like immunoreactivity is reported in several distinct cell types throughout the body of *L. clarki*. FMRFamide-like immunoreactive cell bodies were found in the buccal tentacles, esophageal region and in proximity to the radial nerve cords. Sensory-like cells in the tentacles send processes toward the circumoral nerve ring, while unipolar and bipolar cells close to the radial nerve cords display extensive processes in close association with muscle and other cells of the body wall. Histamine-like immunoreactivity was identified in neuronal somatas located in the buccal tentacles, circumoral nerve ring and in papillae distributed across the body. The tentacular cells send processes into the nerve ring, while the processes of cells in the body wall papillae extend to the surface epithelium and radial nerve cords. Pharmacological application of histamine produced a strong coordinated, peristaltic response of the body wall suggesting the role of histamine in the feeding behavior. Our immunohistochemical data provide evidence for extensive connections between the hyponeural and ectoneural nervous system in the sea cucumber, challenging previously held views on a clear functional separation of the sub-components of the nervous system. Furthermore, our data indicate a potential function of histamine in coordinated, peristaltic movements; consistent with feeding patterns in this species. This study on *L. clarki* illustrates how using a broader range of neurotransmitter systems can provide better insight into the anatomy, function and evolution of echinoderm nervous sytems.

## Introduction

Recent revisions of the deuterostome phylogeny [1], [2] suggest that there are at least 4–6 distinct lineages within the clade and these lineages feature radically different types of nervous system organizations ranging from a true brain and dorsal nerve tube in chordates to diffuse skin nerve nets in Xenoturbella. For Ambulacraria (composed of hemichordates and echinoderms), this diversity is further exemplified by the many larval forms that feature drastically different morphologies from the adults. While the neuroanatomy of the larval nervous system has been extensively studied for a range of echinoderm species (reviewed in [3], [4]), the link and mechanisms of transformation between the larval and adult nervous system remain largely elusive [5].

The echinoderm nervous system primarily consists of a circumoral nerve ring connected to five radial nerve cords that run between the longitudinal and circular muscles of the body. In the majority of echinoderms (except Crinoids), the nervous system has traditionally been described as containing two separate compartments, the ectoneural and hyponeural subsystems, separated by a basement membrane [6], [7]. The ectoneural subsystem comprises the circumoral nerve ring and the thicker, outer part of the radial nerve cords, and has been ascribed both sensory and motor components. The hyponeural subsystem is a thinner, inner layer of the radial nerve cords traditionally thought to control locomotion [6], [7]. Classically, these compartments were viewed as completely separate subsystems with no or limited interactions [8], [9], [10], [11], [12].

Recent studies on larval and adult nervous systems and specifically those of holothurian species have begun to challenge many of these viewpoints of echinoderm neurobiology. These include the discovery of short neural bridges connecting the ectoneural and hyponeural subsystem [13], [14]; and the presence of an extensive network of glial-like cells and chemical synapses, both features thought to be missing from echinoderm nervous systems [15]. Within the class Holothuria (sea cucumbers), the description of a few additional components has significantly expanded our understanding of the echinoderm nervous system. These include description of the enteric nervous system, nerve plexi associated with connective tissues and the structure and circuitry of the tube feet [16], [17], [18], [19]. Finally, developmental studies provide increasing evidence for an ectodermal instead of a mesodermal origin of the hyponeural subsystem as previously assumed [14], [20]. Together, these findings suggest that echinoderm nervous systems may be more comparable to those of other animals and the study of the neuroanatomy of holothurian species promises to provide further insights into the organization and function of the nervous systems of this highly successful deuterostome lineage.

Here, we used two neural markers to reveal the architecture of neuronal elements in the semi-transparent sea cucumber *Leptosynapta clarki*: FMRFamide and Histamine. FMRF, a neuropeptide first characterized in molluscs [21], belongs to the FMRFamide and RFamide-like peptide family (RF-amide), which has been subsequently described in many bilaterian taxa and appears to have a wide range of biological functions [22]. In echinoderms, these peptides have been shown to be involved in neurotransmission and were immunohistochemically detected in the radial nerve cord, the tube feet and apical muscle [23]. In holothuroids, RF-amide peptides have been identified in the intestine [24], [25], the body wall [26], [27] as well as radial nerves and in nerve plexuses of the esophagus [25], [28]. Although there are many open questions about the function of RF-amide peptides in echinoderms, it has proven to be a useful neuronal marker in several echinoderm groups [22].

Histamine (HA) is an amine with a variety of physiological and neuronal functions including signal transduction mechanisms in the visual system of insects [29] and *Daphnia* [30], feeding in molluscs [31], the entrainment of circadian rhythms in mammals [32] as well as the inflammatory response [33]. Few studies, however, have focused on histamine signaling in echinoderms. Smith [34] did find significant amounts of histamine in the gonads and digestive organs of the Asteroid *Luidia clathrata.* And it is also known that histamine can act as an intracellular messenger in echinoderm fertilization [35]. Furthermore, recent evidence shows an involvement of HA in sea urchin metamorphosis [36], [37], [38].

**Figure 1:**
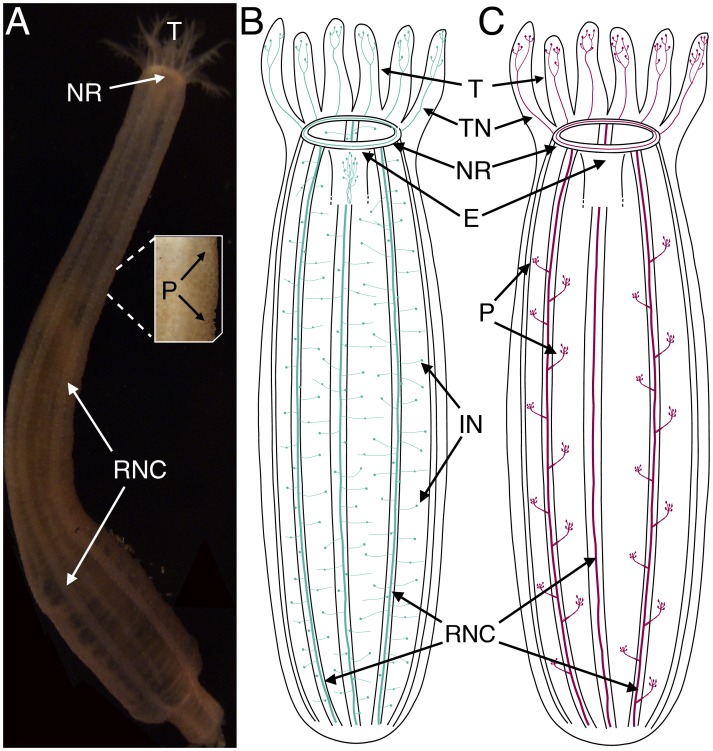
Schematic depiction of holothurian nervous system. A) The semi-transparent *Leptosynapta clarki* is readily found just beneath the sediment during the springtime at False Bay, San Juan Island, WA. Major structures of the echinoderm nervous system are visible through the skin: buccal tentacles (T), circumoral nerve ring (NR), radial nerve cords (RNC) and the dorsal papillae (P). B) FMRFamide-like immunoreactivity was found in processes extending from the nerve ring (NR) into the tentacles (T) (tentacle nerves: TN), in the esophageal region (E) and throughout the body with projections and interneurons (IN) from the radial nerve cords (RNC). C) Histamine-like immunoreactivity was identified in processes extending from the NR and in abundant neurons in the tentacles (T), as well as in processes from the RNCs to sensory-like structures in the papillae (P).

Despite some recent progress in echinoderm neuroanatomy, basic questions about transmitter localization and function in the echinoderm nervous system remain unresolved. Systematic analysis of the distribution of these signaling molecules in the developing and mature nervous systems of echinoderms promises to provide better insights into the function and evolution of the nervous system of these enigmatic organisms (e.g. [15]). With this goal we set out to investigate the distribution of histamine (HA) and FMRFamide in *L. clarki* (Fig. 1). This species is readily found just beneath the sediment during the springtime at False Bay, San Juan Island, WA. *L. clarki* is apodid and uses its feather-like buccal tentacles to push sediment into its mouth for feeding. Peristaltic contractions of the body wall help to move the sediment through the enteric system [39]. As an apodid sea cucumber *L. clarki* do not have tube feet, but they have retained modified podia in the form of dorsal papillae (see Fig. 1). The dorsal papillae have long been attributed a sensory role [6] and from ultrastructural studies in *Holothuria forskali* the function of the dorsal papillae has been confirmed as either mechanosensory or chemosensory [16]. Our immunohistochemical data suggest a role for histaminergic signalling in the ciliated cells that project into the epidermal layer of the papillae, and pharmacological tests with histamine suggest a putative role for histamine in modulating the feeding response. We also identified several previously unknown nerve clusters within the buccal tentacles of the burrowing sea cucumber *L. clarki*, using both histamine and FMRFamide immunohistochemistry. To our knowledge none of these neuronal markers have been fully characterized in this species, and our study provides the first evidence for extensive histamine-like neurons in adult holothurians.

## Materials and Methods

Animals were collected on three separate occasions from False Bay, San Juan Island, WA, USA in early April, May, and June 2008 under the auspices of permits from the State of Washington to Friday Harbor Laboratories. The May and June trips fell during the juvenile recruitment phase [40], allowing comparative analysis of the nervous system structure and behavior of animals from different size classes.

### Immunohistochemistry

The immunohistochemical protocol was identical for adults and juveniles. Excess sediment was carefully removed from each specimen with forceps under a Nikon dissecting scope. Specimens were then relaxed in filtered sea water containing 337 mM MgCl_2_ for 20 minutes at 4°C. Animals were pinned down into Sylgard coated plates with size 000 insect pins and dissected by making one incision running through the buccal nerve ring straight down the body in between two radial nerve cords. Dissected animals were further stretched with pins and then, to prevent contraction during fixation, 4% paraformaldehyde in 1% PBT (1X phosphate buffered saline with 1% Triton X, pH ∼7.4) was slowly introduced to the 337 mM MgCl_2_ solution. Then the volume was completely replaced with fixative and incubated either 4 hours at room temperature or overnight at 4°C. Specimens were then further dissected and transferred to 24-well plates for immunohistochemical processing. Most tissue samples were post-fixed for 20 minutes in cold 100% Methanol at 4°C and then washed six times for sixty minutes each in 1% PBT at room temperature to permeabilize the membrane. However, when muscle fibers needed to be visualized the methanol post-fixation could not be used because it was found to be incompatible with the rhodamine phalloidin secondary stain. Tissues were then incubated in 5% goat serum with 1% PBT overnight at 4°C to block non-specific binding of the primary antibody. Then tissues were transferred to a new well and incubated with primary antibodies for 48–72 hours at 4°C. The primary antibodies used were FMRFamide (1∶1500) [ImmunoStar 20091] and Histamine (1∶1000, (Abcam # ab43870-100; Lot 740889) [38] – for validation of antibody in echinoderm tissues), all diluted in 5% goat serum with 1% PBT. Primary antibodies were washed six times for sixty minutes each with 1% PBT at room temperature and then incubated with secondary antibodies overnight at 4°C. Anti-rabbit and anti-goat secondary antibodies were used at 1∶400 in 4% goat serum with 1% PBT. The next day secondary antibodies were washed off five times for sixty minutes each in 1% PBT at room temperature. Samples were then incubated with secondary stains for 30–45 minutes at room temperature. Secondary stains used included rhodamine phalloidin (1∶400) (Invitrogen) to stain for actin and Draq-5 (1∶1000) (Biostatus, Inc. UK), nuclear stains all diluted in 1% PBT. Secondary stains were washed off three times for five minutes each in 1X PBS at room temperature. Finally slides were mounted in DABCO (1,4-diazabicyclo[2.2.2]octane, Sigma, Canada) and imaged with a confocal microscope. Controls were performed by foregoing the addition of primary antibody. Samples were all imaged on a BioRad Radiance 2000 mounted on a Nikon E800 confocal microscope using appropriate wavelengths. Image analysis and 3-D reconstruction were done in ImageJ [41], [42].

### Pharmacological Tests

All pharmacological tests were performed in blocks of pairs of mature, adult specimens (see Movies S1 and S2). Each trial was recorded with a Sony (DCR-SR72E) Digital Camcorder using the time-lapse recording feature in BTV Pro (http://www.bensoftware.com/) to record for 30 minutes with 0.5-second intervals. Animals were selected to have a similar size and the internal volume of the animal was estimated based on relaxed body length. After the control trial recording, one animal was injected with a volume of histamine that upon dilution in the body cavity would approximate a 200 µM concentration and the other animal was injected with the same volume of filtered seawater. Immediately after injection animals were again recorded using the time-lapse recording feature in BTV Pro for 45 minutes with 0.5-second intervals. Responses were quantified by counting the number of peristaltic movements in each direction and dividing by the total number of minutes in each trial. Scoring was done blindly with reference to treatment state. The effect of histamine on the frequency of directed peristaltic movements was then analyzed with a Model I ANOVA using R, version 2.11.0.

## Results

First, we describe the distribution patterns of HA- and FMRFamide-immunoreactivity in *L. clarki* and compare them to each other as summarized in Fig. 1B,C. Second, we provide evidence that exposure of adult *L. clarki* to histamine increases peristaltic muscular contractions.

### FMRFamide-immunoreactivity (FMRF-IR)

**Figure 2:**
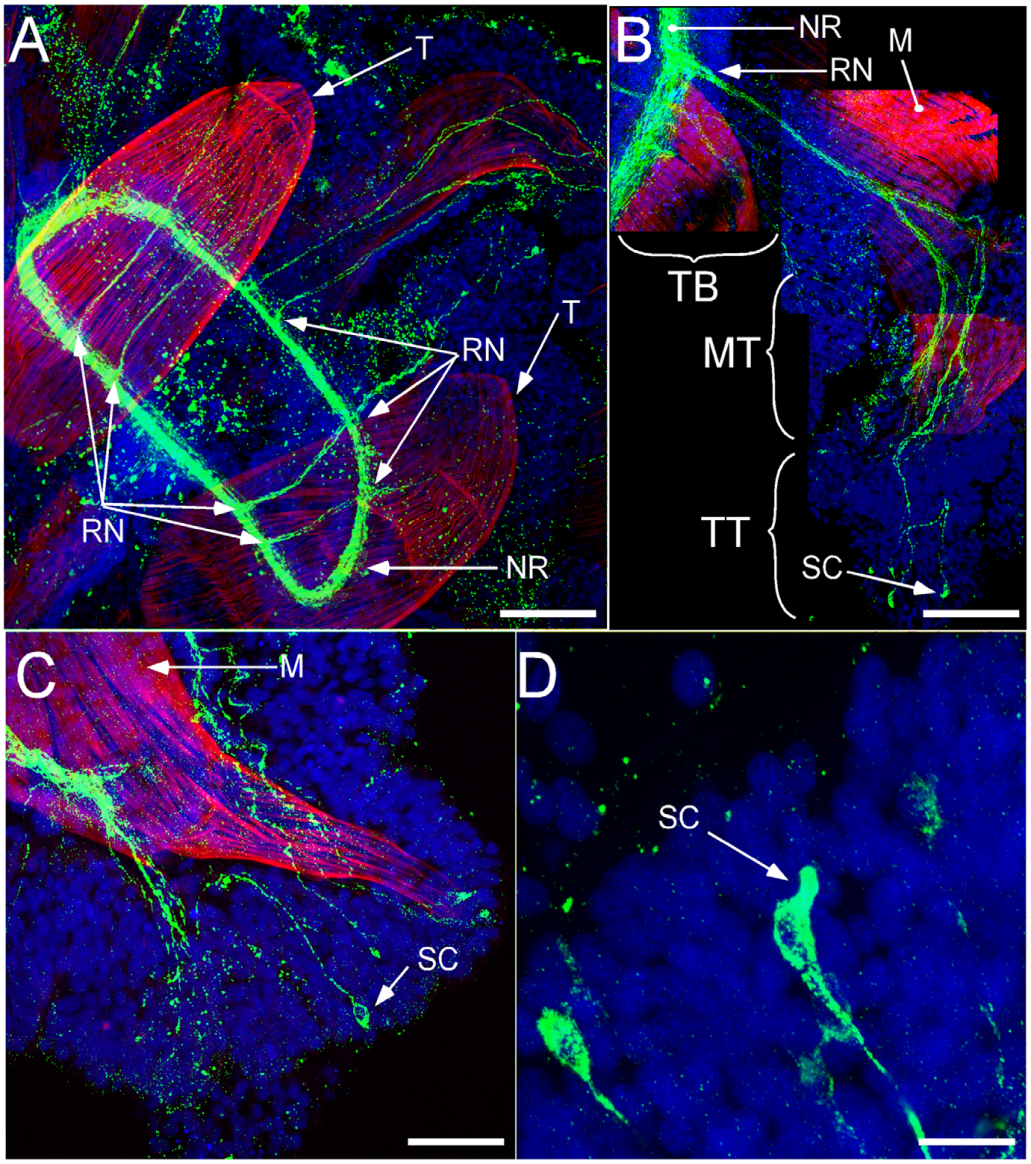
FMRFamide-like immunoreactivity (green) is seen throughout the buccal region of *Leptosynapta clarki* juveniles. Note that some of the staining in the nerve ring is background based on comparisons to control samples (no primary antibody). Red indicates muscle fibers visualized by phalloidin (actin filaments) stain. Nuclei are labeled with Draq-5 and are colored in blue. A) Composite panel of FMRFamide-like immunoreactivity surrounding the circumoral nerve ring (NR) and projecting into each tentacle as two radial nerve extensions (tentacle nerves: TN); scale bar 100 µm B) Composite panel of two tentacles at higher magnification shows the furcation of these extensions from the mid tentacle region (MT) culminating in putative sensory cells visible at the tip of tentacle (TT); scale bar 60 µm C) Higher magnification of one tentacle clearly shows the extension of one putative sensory cell beyond the muscle fiber (red) and into epithelial tissue; scale bar 50 µm (D) further magnification of these cells reveals projections characteristic of sensory cells; scale bar 10 µm.

**Figure 3:**
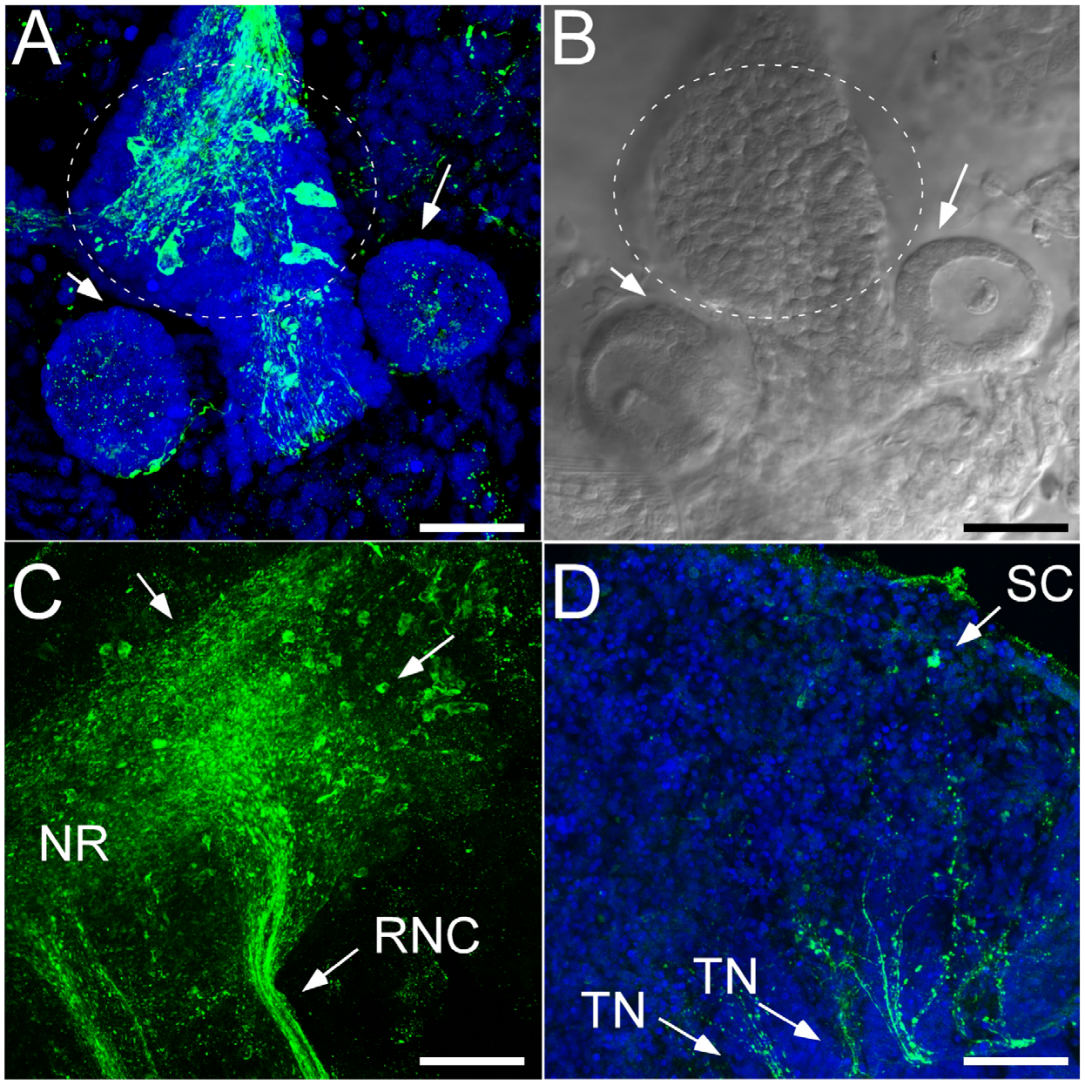
Mouth region and tentacles of adult *Leptosynapta*. A) Dissection of adult tentacle reveals an abundance of FMRFamide-like immunoreactive radial nerve extensions throughout the tentacle and a concentration of putative sensory cells in the esophageal region (circle); scale bar 25 µm B) DIC image corresponding to A. Arrows point to cross-sections of two buccal tentacles. C) Dense aggregation of processes and cell bodies are visible in the esophageal region and the anterior region of the radial nerve cord (RNC) where it meets the circumoral nerve ring (NR). Arrows point to cell bodies; scale bar 50 µm D) FMRFamide-like immunoreactivity in tentacles of adult. Arrows point to sensory cells (SC) in the periphery of the tentacle and their processes projecting to the NR via the TN; scale bar 50 µm. Blue: Nuclear stain using Draq-5, Green: Anti-FMRFamide antibody.

**Figure 4:**
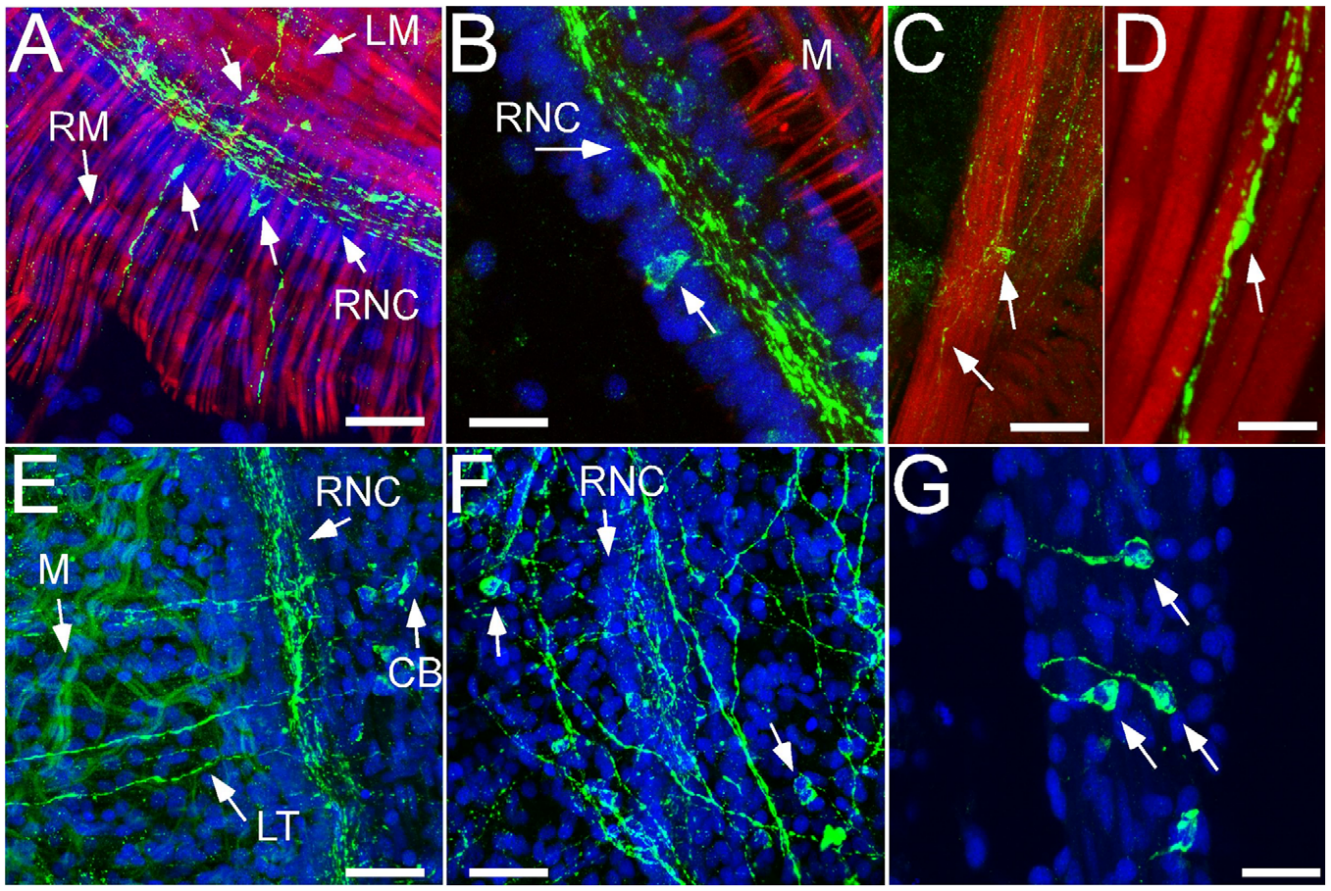
FMRFamide-like immunoreactivity (FMRF-IR) is also found throughout the mid-body and posterior region of adult *Leptosynapta clarki*. A) Close association with interneurons and muscles in proximity of the radial nerve cord (RNC). This section shows both radial (RM) and longitudinal muscles (LM). scale bar 25 µm B) putative interneurons are found intermittently along the RNC and appear associated with axons in the RNC. RM are visible in the background; scale bar 25 µm C,D) high magnification images of a FMRF-IR process associated with a muscle fibre (arrows). No cell bodies are visible in these views; scale bars 25 µm and 10 µm respectively E) FMRF-IR network of axons in proximity of radial nerve cord is also associated with individual cell bodies (CB). In this image, bleed-through fluorescence from dispersed muscle fibers (M) is visible; scale bar 50 µm. F) close-up of network also seen in E. Cell bodies are clearly visible in body wall; scale bar 50 µm. G) at higher magnification these cell bodies do not appear to have the characteristic projections of those seen in the buccal region or other parts of the mid-body. scale bar 25 µm. Blue: Nuclear stain using Draq-5, Green: Anti-FMRFamide antibody.

We detected FMRFamide immunoreactivity (FMRF-IR) in several hundred neurons from the anterior, mid and posterior body of juvenile and adult *L. clarki* (Fig. 1B and Fig. 2–4). Specifically, we identified cell bodies of numerous bipolar neurons. These sensory-like neurons and their processes were abundant in the tentacles, the esophageal region and in proximity of the radial nerve cords. A subpopulation of FMRF-IR cells in the tentacles and the body wall have a distinct shape with similarity to vertebrate sensory neurons. We refer to these cells here as “sensory-like neurons” due to this similarity. Specifically, they have a distinct bipolar organization with apical processes extending towards the epithelium. This staining pattern is most explicit in a small subset of sensory-like cells in the tentacles (Fig. 2B–D). However, FMRF-IR is also found in unipolar cells throughout the midbody and posterior regions as well as bipolar cells in the body wall (Fig. 3–4), possibly indicating a wide diversity of functions of several peptidergic neuronal populations. These neurons appear to be a part of a network with widely distributed cell bodies in proximity to the radial nerve cords (RNCs) and the tentacles. We detected abundant FMRF-IR in what we identified as the ectoneural and hyponeural part of the nervous system (e.g. Fig. 4C). No staining was observed in the basement membrane that separates these two compartments from each other.

#### Anterior region

We identified bundles of axons connecting cells in the tentacles to the circumoral nerve ring (Fig. 2). Due to the thickness of the tissue it was not possible to follow all projections to their final target, but Fig. 2A, B and C show the complete projection pattern for one tentacle. The tips of the tentacles are characterized by having distinct FMRF-IR, sensory-like cells (Fig. 2D). Cytoplasmic protrusions of these cells penetrate the epithelial layers and apparently they are in contact with the environment as illustrated in Fig. 3D. Some of the processes connecting these sensory-like cells to the nerve ring showed varicosities, but they were less dense then those labeled with histamine (see below). FMRF-IR also identified bundles of sensory-like cells with elongated, apical-neuronal branches in the esophageal region (Fig. 3A). The morphology of these cells is distinct from those found in the tentacles, as many of these cells have subtle, basal neuronal branches that extend toward the esophageal cavity (Fig. 3A,B). Dense aggregations of neurites and cell bodies are also visible directly anterior to the RNC in the esophageal region (Fig. 3C) and these cell bodies do not appear to have extensive neuronal processes as found in the tentacles.

#### Mid-body and posterior region

We detected axons and dispersed neurons with multiple connections to other FMRF-IR neurons along the RNCs (Fig. 4). We view these neurons as putative interneurons based on their multi-polar organization. Analysis of 3-D reconstructions of these regions based on confocal stacks revealed that while some putative interneurons in proximity of the RNCs are connected to the axons of the RNCs (Fig. 4A,B), others are not (Fig. 4G). These interneurons likely connect to other types of neurons within this extensive network (see Fig. 4E,F) but these targets will need to be identified using antibodies to other neurotransmitters and/or peptides. Using phalloidin stain for muscle fibers we were able to document a close association of some FMRF-IR neurons with muscle suggesting motor functions for this subpopulation (Fig. 4C,D).

### Histamine-like Immunoreactivity (HA-IR)

**Figure 5:**
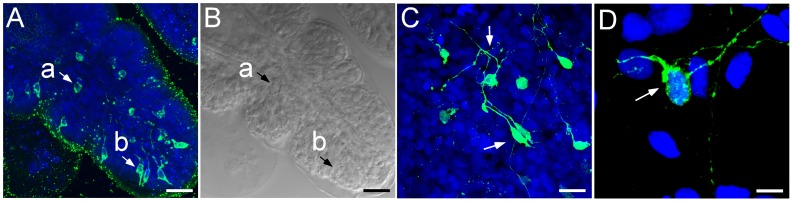
Histaminergic-like immunoreactivity found in the buccal tentacle region of juvenile *Leptosynapta clarki*. Cell bodies are clearly labeled with histamine and are morphologically similar to FMRFamide-like neurons in the tentacles (see Fig. 3). A) individual cells with typical, sensory-like projections are visible at the epithelial surface (b) putative neurons without projections are found in deeper tissue layers (a). In this preparation only few processes are visible. scale bar 50 µm. B) corresponding individual DIC optical section of Fig. 5A shows the position of the two distinct cell types in the tentacle. scale bar 50 µm. C) Higher magnification of the sensory-like tentacle neurons; scale bar 25 µm. D) Close-up of putative sensory neuron with characteristic projection into distal tentacle region. scale bar 10 µm. Blue: Nuclear stain using Draq-5, Green: Anti-histamine antibody.

**Figure 6:**
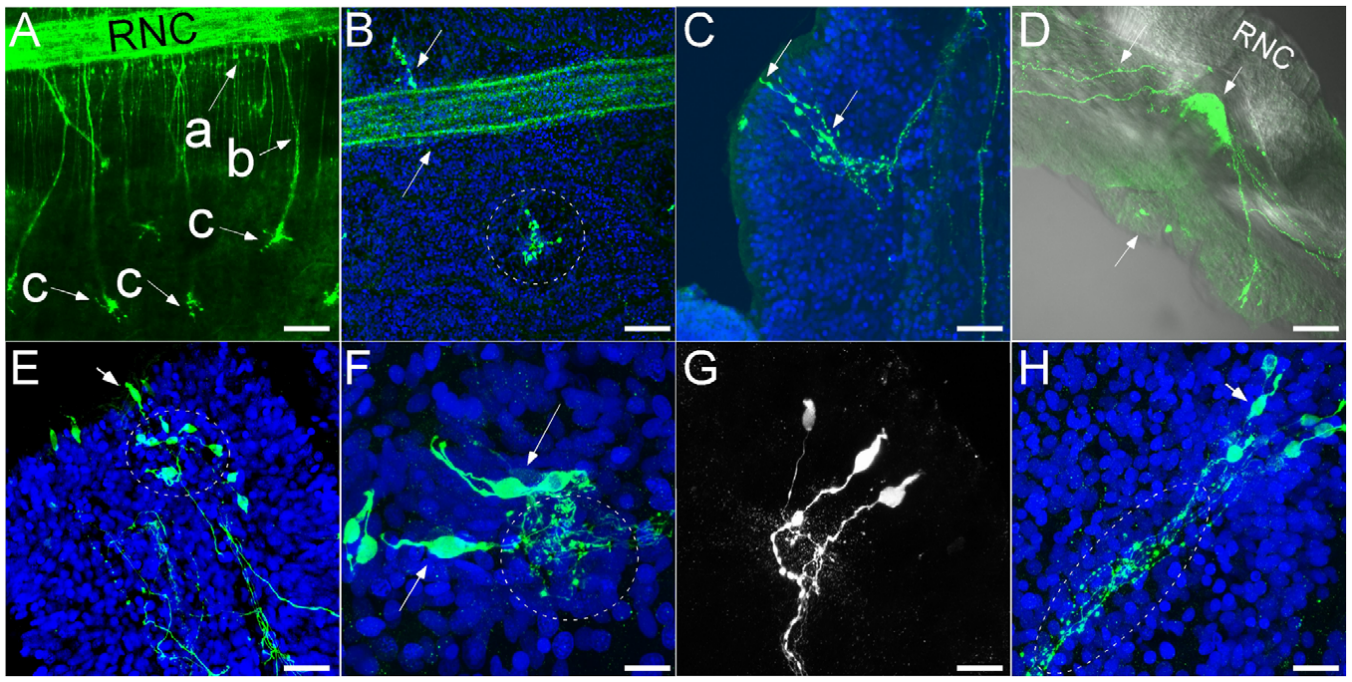
Histaminergic-like immunoreactivity (HA-IR) seen throughout the mid-body region of *Leptosynapta clarki* juveniles and adults. A) high concentration of HA-IR neurons in the center of the body wall papillae (c). These neurons project to the radial nerve cord (RNC) (b) and connect in dense bundles (a) (see also D for cross section of RNC); scale bar 100 µm. B) Higher magnification view of RNC and two body wall papillae; ); scale bar 50 µm. Neurons in the papillae have distinct sensory protrusions that reach to the outside of the epithelial layer (C and E; scale bars 50 µm). D) cross section of RNC with projections from body wall papillae sensory neurons to RNC; HA-IR is combined with DIC image which shows the association of histaminergic neurons with latitudinal muscle fibers; scale bar 50 µm. F:G) Sensory neurons of body wall papillae form distinct glomeruli-like structure indicating sites of dense synaptic connections (E). This glomeruli-like structure is visible in G and H; scale bar 25 µm.

In contrast to the remarkable diversity of FMRF-IR cells, histamine labeling is primarily associated with distinct populations of sensory-type neurons in individual papilla that line the body (see Fig. 1A,C and Fig. 5–6). We found dense aggregations of 3–10 HA-IR neurons in the papillae of the body wall of *L. clarki* in both juveniles and adults which form a remarkable glomeruli-type organization (see below). As was the case for FMRF-IR, we did not find evidence for a separation of the ectoneural and hyponeural nervous system. HA-IR neurons appear to be present in both parts and connections between these systems appear to be present as well (e.g. Fig. 6D). We also did not find HA-IR in the basal membrane.

#### Anterior region

We identified an abundance of putative histaminergic neurons in the buccal tentacles and in the region of the circumoral nerve ring resembling putative sensory cells (Fig. 5A,B). Tentacular neurons project axons to the circumoral nerve ring and show a distinct branching pattern forming a compact glomeruli-like region with potential sites of dense synaptic connections (Fig. 5C). These neurons appear to project directly to the circumoral nerve ring (not shown).

#### Mid-body, posterior region and body wall

HA-IR neurons in the body wall are exclusively concentrated in body-wall papillae (Fig. 6). In each papilla, 3–10 of these neurons are grouped together. They also have distinctive morphologies that resemble olfactory-type or taste-type sensory neurons with a short neurite exposed to the environments and basal neurites that cluster together forming a compact glomeruli-like region (Fig. 6F,H, G). These glomeruli-like regions display abundant varicosities with potential sites of dense synaptic connections (Fig. 6C,F,H) and project to the RNC. In some cases we were also able to identify extensions into the epithelium that appear to follow latitudinal muscle fibers (Fig. 6D).

### Pharmacological Tests with HA

**Figure 7:**
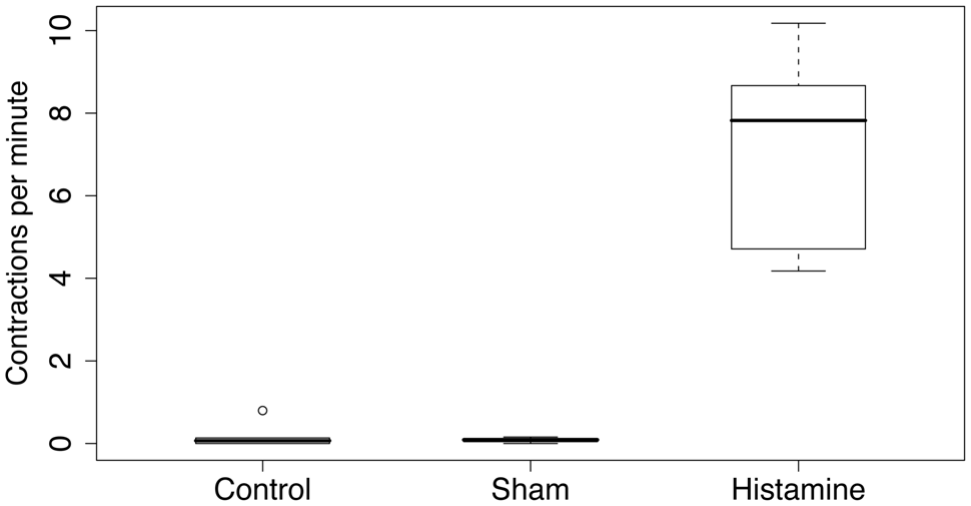
*Leptosynapta clarki* adults respond to histamine injection (200 µM) with a drastic increase in anterior to posterior directed peristaltic contractions. Thick lines in the middle of the boxplots show the median number of anterior to posterior contractions per minute and the whiskers delineate the interquartile range. Control and sham specimen (filtered seawater, 200 µM) rarely displayed anterior to posterior peristalsis, while histamine injected animals consistently responded with rapid increases in the frequency of anterior to posterior peristalsis (F(1,8) = 46.8874, *P* = 0.001).

Considering the very unique pattern of HA-IR, we performed pharmacological experiments to test a functional significance of HA in the sea cucumber behavior. Injection of histamine (see methods; Movies S1 and S2) produced an immediate and significant increase in anterior to posterior peristalsis (F(1,8) = 46.8874, *P* = 0.001). Specifically, all animals injected with histamine increased the frequency of anterior to posterior contractions about eight times more often per minute than control and sham control animals (Fig. 7). We did not test the effects of the RFamide–class of neuropeptides, since this family represents a very diverse group of secretory peptides and no molecular data are currently available for this species.

## Discussion

The paucity of information about echinoderm nervous systems motivated this detailed immunohistochemical survey of neurotransmitter distribution in the nervous system of the holothurian *Leptosynapta clarki*. To our knowledge the FMRF and HA antibodies used in this study have never been used on any holothuroids. However, other RFamides with similar structures have been tested. For example, the nervous systems of *Holothuria glaberrima* and *Stichopus japonicus* were studied using antibodies against different SALMF-amides [18], [25], [43], [44], [45].

### Comparison of FMRFamide and HA Immunoreactivity

Traditionally, holothurian radial nerves are thought to be subdivided into distinct ectoneural and hyponeural regions and connected by small neural bridges [14]. This subdivision presents the opportunity for differential signal propogation between these regions. Based on our observations, we find both FMRFamide-immunoreactivity (FMRF-IR) and HA-immunoreactivity (HA-IR) throughout both regions, and we were unable to find an anatomical separation of these two sub-compartments based on these two neurotransmitter labelings (see for example Fig. 4C and Fig. 6D).

Although we found both FMRFamide- and HA- immunoreactivity in sensory-like cells in the tentacles, we are confident that these sensory cells belong to at least two anatomically and functionally separate neuronal populations. As both antibodies were generated from the same animal host we were unable to perform double-labeling, but we consistently found more immunoreactivity in the tentacular neurons with the HA antibody than with FMRFamide antibody (see for example Fig. 2C and Fig. 5A). We also did not find HA-IR neurons that are dispersed in the body wall, as was the case for FMRF-IR neurons, and conversely no FRMF-IR neurons were found in the papillae. Overall, HA-IR was restricted to sensory-like cells and projections in the tentacles and body-wall papillae (Fig. 1C), whereas FMRF-IR appeared in numerous, morphologically diverse cell types distributed throughout the tentacles, body wall and esophageal region (Fig. 1B) suggesting a wide spectrum of functions for the groups of peptidergic neurons revealed with this marker.

Comparing our FMRF-IR patterns to those in the sea cucumber *Holothuria glaberrima* and in other holothurian species [e.g. 18,28,44,45] reveals similarities in the innervation of the longitudinal muscle layer, the esophageal region and the radial nerve cord (RNC). However, the FMRFamide-antibody we used appears to stain significantly more neuronal somatas than previously reported.

Our data on the distribution of HA-IR and FMRF-IR neurons in *L. clarki* confirm previous findings that a clear separation between the ectoneural and hyponeural nervous system does not exist [14]. Both neuronal systems show extensive connections between epithelial and muscular components. This is in contrast to previous suggestions that these compartments are separated from each other (e.g. [46]). Evidence for a lack of such a separation has also emerged recently from morphological studies of other sea cucumber species (e.g. [14]). If there are truly sensory neurons projecting from the hyponeural system, then the subsystems functional distinction becomes blurred as well.

### Mutable Connective Tissue/Muscle Contraction

In contrast to vertebrates, the majority of muscles in adult echinoderms consist of non-striated fibers [47], [48] and the mechanisms of muscle tone regulation in echinoderms have been extensively studied (for review see [49]). For example, it has been shown that nitric oxide and SALMFamides cause relaxation in certain muscle types. In addition to these muscle types, it has been proposed that echinoderms use a mutable or catch-connective tissue in the body wall that can control the rigidity of the structure [47], [49], [50]. Recent investigations suggest that this mutable, connective tissue also involves muscle cells [51].

Pharmacological tests in sea cucumber species have shown that several, small peptides can cause contraction and relaxation of the body wall [26], [27]. Several researchers have also reported muscular response to histamine in Holothuroidea. For instance, isolated preparations of cloacal muscle from *Cucumaria frondosa* normally display rhythmic contractions. Application of histamine to this cloacal muscle preparation increases the tone of contractions while simultaneously decreasing the rate and amplitude of contraction [52]. Histamine was also found to induce contractions of the respiratory trees and relaxation of longitudinal muscle of *Holothuria* sp. [53] as well as to induce contractions of the contractor muscle of *Cucumaria japonica* [54]. But histamine application produced no effects on the longitudinal muscles of *Holothuria grisea* [55] nor on isolated protractor muscles of *Echinometra lucunter* [56]. Histamine does cause reproducible relaxation of tube foot muscle in *Asterias* amurensis, indicating a possible role of histamine as a neuromodulator of tube foot function [57]. Together these results suggest a neuromuscular control mechanism in echinoderms.

In *L. clarki*, both FMRF-IR and HA-IR appear to be associated with muscle fibers (e.g. Fig. 4C,D and Fig. 6D). This is interesting in light of the comparative information above as it suggests that these transmitters may be functionally involved in controlling muscle contractions. While we were unable to test this hypothesis for FMRFamide, we did find that exposure of *L. clarki* adults (Fig. 7) and juveniles (data not shown) to HA led to an increased frequency of peristaltic feeding behavior. During feeding, sea cucumbers push sediment into their espophagus with their buccal tentacles [39]. Once in the esophagus, anterior to posterior contractions are required to further move sediment through the digestive system. We also identified HA-IR neurons throughout the radial nerve cord. From there, projections extend into the epithelium and into the circular and longitudinal muscle. In summary, the distribution of histaminergic-like cells and the strong response to application of the drug suggest an important role for histamine in motor aspects of feeding. However, more functional data is needed to determine if histamine controls the feeding response or is merely involved in modulating the feeding response.

### Putative Sensory Functions of Histamine

Histamine is a widely distributed signaling molecule across animal phyla and across tissue types [58], with relatively high abundance in organs frequently in contact with the environment such as the skin [59]. Mettrick and Telford (1965) were the first to identify the presence of histamine in Holothuria, using biological assays (e.g. response of the ileum of guinea pig and response of the blood pressure of dog). They found appreciable levels of histamine in the intestine and respiratory trees of *Holothuria* sp., but not in the body wall or tentacles of this species [60]. Lorenz et al. [58] also detected modest amounts of histamine in the intestinum of *Holothuria tubulosa* using fluorescence spectrometry. Our study complements this work by providing evidence, for the first time, of HA-IR in neurons in the sea cucumber tentacles. These HA-IR neurons, in the tentacles and dorsal papillae, feature an intriguing similarity to vertebrate sensory neurons with clear cytoplasmic protrusions of the cell body into the outermost epithelium and with processes displaying extensive varicosities. While these structural characteristics are indicative of chemosensory cells [16], other sensory modalities cannot be ruled out without more functional tests.

Histamine has now been identified as an important signaling molecule in sea urchin metamorphosis, both as an environmental cue [36], [37], [61], [62] and as a modulator of metamorphic competence [38]. Published data also shows that this neurotransmitter plays a critical modulatory role in sea urchin fertilization. Specifically, sea urchin eggs express a histamine receptor on their cell surface, and upon fertilization the histamine H1 receptor activates the NO pathway in order to maintain Ca^2+^ -signaling [35], [63], [64]. In both of these capacities, histamine appears to be an essential modulator of internal physiological functions in response to environmental input. The present results suggest that additional sensory and motor roles for the histaminergic system should be explored.

Important questions about the separation of echinoderm nervous subsystems and the presence and function of neurotransmitter systems remain unclear. Still, insights into the structure and function of these systems can make critical contributions to our understanding of the evolution of nervous systems in animals. This study in *L. clarki* supports the emerging consensus that echinoderm nervous subsystems are less separated than once thought, and provides further support for a diverse role of RFamides in the echinoderm nervous system. Finally, this study provides evidence for a novel involvement of histamine in the sensory system of an adult echinoderm. Taken together, these insights offer tantalizing hope that the overall picture of the echinoderm nervous system is becoming more clear and that with more data many of the enigmas will be solved.

## Supporting Information

Movie S1 **Control recording of two adult** ***L. clarki*** **in a divided petri dish filled with sea water.** Animals were selected to have a similar size and the internal volume of the animal was estimated based on relaxed body length. Time-lapse recording captured behavior for 30 minutes with 0.5-second intervals and the frequency of directed peristaltic contractions was counted for each animal (N = 8).
(MP4)

Movie S2 **Experimental recording of the same two adult** ***L. clarki*** **in a divided petri dish filled with sea water.** After the control recording, one animal (on the left) was injected with a volume of histamine that upon dilution in the body cavity would approximate a 200 µM concentration and the other animal (on the right) was injected with the same volume of filtered seawater. Injection of histamine (on the left) clearly increases the frequency of anterior-to-posterior peristaltic contractions. Blind scoring of additional trials on different animals showed that this histamine-induced change in the direction of peristaltic contraction is highly repeatable (see Fig. 7).
(MP4)
